# Dynamic optimization of distributed biological systems using robust and efficient numerical techniques

**DOI:** 10.1186/1752-0509-6-79

**Published:** 2012-07-02

**Authors:** Carlos Vilas, Eva Balsa-Canto, Maria-Sonia G García, Julio R Banga, Antonio A Alonso

**Affiliations:** 1 BioProcess Engineering Group, IIM-CSIC, Eduardo Cabello, 6, 36208 Vigo, Spain

**Keywords:** Dynamic optimization, Distributed biological systems, Reduced order models, Global optimization methods, Hybrid optimization methods, Pattern formation and control

## Abstract

**Background:**

Systems biology allows the analysis of biological systems behavior under different conditions through *in silico* experimentation. The possibility of perturbing biological systems in different manners calls for the design of perturbations to achieve particular goals. Examples would include, the design of a chemical stimulation to maximize the amplitude of a given cellular signal or to achieve a desired pattern in pattern formation systems, etc. Such design problems can be mathematically formulated as dynamic optimization problems which are particularly challenging when the system is described by partial differential equations.

This work addresses the numerical solution of such dynamic optimization problems for spatially distributed biological systems. The usual nonlinear and large scale nature of the mathematical models related to this class of systems and the presence of constraints on the optimization problems, impose a number of difficulties, such as the presence of suboptimal solutions, which call for robust and efficient numerical techniques.

**Results:**

Here, the use of a control vector parameterization approach combined with efficient and robust hybrid global optimization methods and a reduced order model methodology is proposed. The capabilities of this strategy are illustrated considering the solution of a two challenging problems: bacterial chemotaxis and the FitzHugh-Nagumo model.

**Conclusions:**

In the process of chemotaxis the objective was to efficiently compute the time-varying optimal concentration of chemotractant in one of the spatial boundaries in order to achieve predefined cell distribution profiles. Results are in agreement with those previously published in the literature. The FitzHugh-Nagumo problem is also efficiently solved and it illustrates very well how dynamic optimization may be used to force a system to evolve from an undesired to a desired pattern with a reduced number of actuators. The presented methodology can be used for the efficient dynamic optimization of generic distributed biological systems.

## Background

Living organisms can not be understood by analyzing individual components but analyzing the interactions among those components [1,2]. In this regard, many efforts are being devoted to formulate mathematical models that enable the possibility of developing and testing new hypotheses about biological systems.

In recent years the use of optimization techniques for the purpose of modeling has attracted substantial attention. In particular, mathematical optimization is the underlying hypothesis for model development in, for example, flux balance analysis [3], or the activation of metabolic pathways [4-6] and is at the core of model identification, including parameter estimation and optimal experimental design [7].

Despite the success of modeling efforts in systems biology, the truth is that only in few occasions those models have been used to design or to optimize desired biological behaviors. This may be explained by the difficulty on formulating and solving those problems but also in the limited number of software tools that may be used for that purpose [8]. In this regard, the recently developed toolbox DOTcvpSB [9] can handle the dynamic optimization of lumped systems (described in terms of ordinary differential equations), such as those related to biochemical processes (see the reviews by Banga et al. [7,8,10] and the works cited therein), or to biomedical systems [11-16].

It should be noted, however, that many biological systems of interest are being modelled by sets of partial differential equations (PDE). This is particularly the case of reaction diffusion waves in biology (see the recent review by [17]) or spatial organization in cell signaling [18]. The scarce works related to the optimization of this type of systems [19,20] reveal that the problem presents significant computational and conceptual challenges due mainly to the presence of suboptimal solutions and to the computational cost associated to the simulation and, thus, the optimization.

The use of global optimization techniques provides guarantees, at least in a probabilistic sense, of arriving to the global solution. Unfortunately the price to pay is the number of cost function evaluations and the associated computational cost, which increase exponentially with the number of decision variables. This aspect is particularly critical for PDE systems as they are usually solved with spatial discretization techniques (e.g. finite element or the finite differences methods) and the result is a large scale dynamic system whose simulation may take from several seconds to hours.

In this concern, the use of surrogate models has been proposed as the alternative to reduce total computation times. The most promising techniques based on kriging or radial basis functions have been incorporated to global optimization solvers [21-23]. However these methodologies do not integrate any knowledge about the system being optimized, i.e. models are treated as blackboxes. Alternatives for PDE systems rely on the application of reduced order modeling techniques which take into account the phenomena of interest. In particular the use of the proper orthogonal decomposition (POD) approach has demonstrated to be an excellent candidate for simulation, optimization and control [24-26].

This work presents the application of hybrid optimization techniques for the solution of complex dynamic optimization problems related to biological applications. Particular emphasis is paid to the efficiency and robustness of the proposed methodologies. In this regard, the use of a hybrid global-local methodology together with a control refining technique is proposed. In addition, the POD technique is used to reduce the dimensionality (and thus the computational effort) of the original distributed (full scale) models.

To illustrate the usage and advantages of the proposed techniques two challenging case studies will be considered. The first is related to bacterial chemotaxis and considers the achievement of two different objectives as formulated in [19]. In addition, a second dynamic optimization problem related to the FitzHugh-Nagumo (FHN) model [27,28], which describes a number of important physiological processes, such as the heart behavior, is formulated and solved.

## Methods

### Dynamic optimization problem formulation

Dynamic optimization, also called open loop optimal control (OCP), considers the computation of a set of time-dependent operating conditions (usually called controls) which optimize a certain performance index of the dynamic behavior of the biological system, subject to a set of constraints. This problem can be mathematically formulated as follows: *find***u**( *t*) *along t *∈[ *t*_0_,*t*_*f*_]*to minimize (or maximize) the performance indexJ*:

(1) $$J=\phi (x(\xi ,t_{f}),y(t_{f}),t_{f})+\int_{t_{0}}^{t_{f}}L(x(\xi ,t),y(t),u(t),\xi ,t)\text{dt}$$

where *ξ* are the spatial variables, *t* the time and $u(t)={\left[u_{1}(t),\ldots ,u_{c}(t)\right]}^{T}$ is the vector of control variables. *ϕ *(Mayer term) and *L* (Lagrangian term) are scalar functions assumed to be continuously differentiable with respect to all their arguments. The state variables are split into two subsets: those distributed in space $x(\xi ,t)={\left[x_{1}(\xi ,t),\ldots ,x_{s1}(\xi ,t)\right]}^{T}$ and those which depend only on time $y(t)={\left[y_{1}(\;t),\ldots ,y_{s2}(t)\right]}^{T}$.

A given number of constraints must be considered when solving optimal control problem (1). These may be classified in three main groups: 

· the system dynamics that, for the general case of distributed process systems, can be represented as a set of partial and ordinary differential equations (PDEs) of the form:

(2) $$\frac{\partial x}{\partial t}=\nabla \cdot \left(k\nabla x\right)-\nabla \cdot \left(vx\right)+f(x,y)+u$$

(3) $$\frac{dy}{dt}=g(x,y,u)$$

with ∇ being the gradient operator and *f*( **x**, **y**, **u**) and *g*( **x**, **y**, **u**) two given (possibly nonlinear) functions which may represent for instance chemical reactions. This system must be completed with appropriate initial and boundary conditions which, for the general case, read as follows:

(4) n·∇x(B,t)=p−qx(B,t)

where **n** is a unit vector pointing outwards the boundary B while *p* and *q* are given (possibly nonlinear) functions. Different types of boundary conditions can be derived from equation (4). For instance homogeneous Neumann conditions are obtained by fixing *p *= *q *= 0. On the other hand, setting $p=hx_{\infty }$ and *q *= *h*, with $x_{\infty }$ being the value of the **x **in the surrounding media, Robin boundary conditions are recovered.

· the bounds for the control variables:

(5) $$u^{L}\leq u(t)\leq u^{U}$$

· and possibly other equality or inequality constraints, which must be satisfied over the entire process time (path constraints) or at specific times (point constraints), being a particular case of the later the final time constraints which must be satisfied at final time. These constraints can be expressed as:

(6) $$c\left(x(\xi ,t),y(t),u(t),t\right)\leq 0$$

(7) $$c\left(x(\xi ,t_{k}),y(t_{k}),u(t_{k}),t_{k}\right)\leq 0$$

where *t*_*k*_ is a time point, being the final time *t*_*f*_, a particular case.

### Numerical methods

#### Numerical methods for the simulation

Many biological systems of interest exhibit a nonlinear dynamic behavior which makes the analytical solution of models representing such systems rather complicated, if not impossible, for most of the realistic situations. In addition to nonlinearity, these processes may present a spatially distributed nature. As a consequence they must be described using PDEs which, in turns, makes the analytical approach even more difficult. Numerical techniques must be, therefore, employed to solve the model equations.

Most of numerical methods employed for solving PDEs, in particular those employed in this work, belong to the family of *methods of weighted residuals* in which the solution of the PDE system (2) is approximated by a truncated Fourier series of the form^a^[29]:

(8) $$x(\xi ,t)\approx \overset{N}{\underset{i=1}{\sum }}m_{i}(t)\phi_{i}(\xi )$$

Depending on the selection of the *basis functions ϕ*_*i*_(*ξ*) different methodologies arise. In this work, two groups will be considered: those using locally defined basis functions as it is the case in classical techniques like the finite difference method or the finite element method and those using globally defined basis functions.

##### *Methods using local basis functions*

The underlying idea is to discretize the domain of interest into a (usually large) number *N* of smaller subdomains. In these subdomains local basis functions, for instance low order polynomials, are defined and the original PDE is approximated by *N* ordinary differential equations (ODE). The shape of the elements and the type of local functions allow distinguishing among different alternatives.

Probably the most widely used approaches for this transformation are the finite difference and the finite element methods. The reader interested on an extensive description of these techniques is referred to the literature [29-31]. Both of these methods have been successfully applied in the context of dynamic optimization [19,32].

However it must be highlighted that in many biological models, especially those in 2D and 3D, the number of discretization points (*N*) to obtain a good solution might be too large for their application in optimization. Methods using global basis functions, which will reduce the computational effort, constitute an efficient alternative.

##### *Methods using global basis functions*

Different techniques like the eigenfunctions obtained from the Laplacian operator, Chevyshev or Legendre polynomials, among others have been considered over the last decades - see [33] and references therein for a detailed discussion -. Probably the most efficient order reduction technique is the *proper orthogonal decomposition* (POD) [34] and because of this, it will be chosen in this work to obtain the reduced order models. In this approach each element *ϕ*_*i*_(*ξ*) of the set of basis functions in (8) is computed off-line as the solution of the following integral eigenvalue problem [34-36]:

(9) $$\lambda_{i}\int_{V}R(\xi ,\xi^{\prime \prime })\phi_{i}(\xi^{\prime })d\xi^{\prime }=\phi_{i}(\xi )$$

where *λ*_*i*_corresponds with the eigenvalue associated with each global eigenfunction *ϕ*_*i*_. The kernel *R*( *ξξ*^*′*^) in equation (9) corresponds with the two point spatial correlation function, defined as follows:

(10) $$R(\xi ,\xi^{\prime })=\frac{1}{\ell }\overset{\ell }{\underset{j=1}{\sum }}x(\xi ,t_{j})x(\xi^{\prime },t_{j}).$$

with *x*( *ξt*_*j*_) denoting the value of the field at each instant *t*_*j*_and the summation extends over a sufficiently rich collection of uncorrelated snapshots at *j *= 1,⋯, *ℓ*[34]. The basis functions obtained by means of the POD technique are also known as empirical basis functions or POD basis.

The dissipative nature of this kind of systems makes that the eigenvalues obtained from Eqn (9) can be ordered so that *λ*_*i *_≤* λ*_*j*_ for *i *<*j*, furthermore $\lambda_{n}\to \infty$ as n→∞. This property allows us to define a finite (usually low) dimensional subset *ϕ*_*A*_=*ϕ*_1_*ϕ*_2_,…,*ϕ*_*N*_ which captures the relevant features of the system [35,37]. The number of elements (*N*) in this subset is usually chosen using a criteria based on the energy captured by the POD basis. Such energy is connected to the eigenspectrum ${\left\{\lambda_{i}\right\}}_{i=1}^{\ell }$ or, to be more precise, to the inverse of the eigenvalues ${\left\{\mu_{i}\right\}}_{i=1}^{\ell }$ with $\mu_{i}=\lambda_{i}^{-1}$ as follows:

(11) $$E(\%)=100\times \frac{\overset{N}{\underset{i=1}{\sum }}\mu_{i}}{\overset{\ell }{\underset{i=1}{\sum }}\mu_{i}}$$

In order to compute the time dependent coefficients in Eqn (8), the original PDE system (2) is projected onto each element of the POD basis set. In this particular case, such projection is carried out by multiplying the original PDE by each *ϕ*_*i*_and integrating the result over the spatial domain, this is:

(12) $$\begin{array}{lll} \int_{V}\phi_{i}\frac{\partial x}{\partial t}d\xi = & \int_{V}\phi_{i}\left(\nabla \cdot \left(k\nabla \right)-\nabla \cdot v\right)\text{xd}\xi +\int_{V}\phi_{i}\text{fd}\xi \; & \;\\ & +\int_{V}\phi_{i}\text{ud}\xi ;\;i=1,\ldots ,N\; \end{array}$$

Substituting the Fourier series approximation (8) into Eqn (12) leads to:

(13) $$\begin{array}{lll} \;\int_{V}\phi_{i}\overset{N}{\underset{j=1}{\sum }}\phi_{j}\frac{dm_{j}}{\text{dt}}d\xi = & \overset{N}{\underset{j=1}{\sum }}m_{j}\int_{V}\phi_{i}\left(\nabla \cdot \left(k\nabla \right)-\nabla \cdot v\right)\phi_{j}d\xi \; & \;\\ & +\int_{V}\phi_{i}\text{fd}\xi +\int_{V}\phi_{i}\text{ud}\xi \; \end{array}$$

The basis functions obtained from (9) are orthogonal and can be normalized so that:

(14) $$\int_{V}\phi_{i}\phi_{j}d\xi =\left\{\begin{array}{lll} 1 & \mathrm{if} & i=j\\ 0 & \mathrm{if} & i\neq j \end{array}\right)$$

Therefore, Eqn (13) can be rewritten as:

(15) $$\frac{dm_{i}}{dt}=P_{i}m_{A}+F_{i}+U_{i}\;\text{for}\;i=1,\ldots ,N$$

where *P*_*i*_ is a row vector of the form $P_{i}=\int_{V}\phi_{i}$$\left(\nabla \cdot \left(k\nabla \right)-\nabla \cdot v\right)\phi_{A}d\xi$with $\phi_{A}={\left[\phi_{1},\phi_{2},\ldots ,\phi_{N}\right]}^{T}$, while $F_{i}=\int_{V}\phi_{i}\text{fd}\xi$, $U_{i}=\int_{V}\phi_{i}\text{ud}\xi$. *m*_A_ corresponds with the following column vector $m_{A}={\left[m_{1},m_{2},\cdots \;,m_{N}\right]}^{T}$. Expression (14) can be rewritten in matrix form as follows:

(16) $$\frac{dm_{A}}{dt}=P_{A}m_{A}+F_{A}+U_{A}$$

where *P*_*A *_= [*P*_1_;*P*_2_;…;*P*_*N*_], $F_{A}={\left[F_{1},F_{2},\ldots ,F_{N}\right]}^{T}$ and $U_{A}={\left[U_{1},U_{2},\ldots ,U_{N}\right]}^{T}$. Initial conditions for solving Eq (15) are obtained by projecting the original initial conditions *x*( *ξ*,0) over the basis functions, this is $m_{A}(0)=\int_{V}\phi_{i}x(\xi ,0)d\xi$. At this point the basis functions *ϕ*_*A *_are known from Eq (9) while time dependent coefficients are computed by solving Eq (15), therefore the approximation of the original field *x* can be recovered by applying Eqn (8), this is $x\approx \tilde{x}=\phi_{A}m_{A}$. It is important to highlight that the number of elements *N* in the basis subset *ϕ*_A_ can be increased to approximate the original state *x* with an arbitrary degree of accuracy.

#### Dynamic optimization methods

There are several alternatives for the solution of dynamic optimization problems from which the direct methods are the most widely used. These methods transform the original problem into a non-linear programming (NLP) problem by means of complete parameterization [38], multiple shooting [39] or control vector parameterization [40] methods. Basically, all of them are based on the use of some type of discretization and approximation of either the control variables or both the control and state variables. The three alternatives basically differ in: the resulting number of decision variables, the presence or absence of parameterization related constraints and the necessity of using an initial value problem solver.

While the complete parameterization or the multiple shooting approaches may become prohibitively expensive in computational terms, the control vector parameterization approach allows handling large-scale dynamic optimization problems, such as those related to PDE systems, without solving very large NLPs and without dealing with extra junction constraints [32].

The control vector parameterization proceeds dividing the process duration into a number of elements and approximating the control functions typically using low order polynomials. The polynomial coefficients become the new decision variables and the solution of the resulting NLP problem (outer iteration) involves the system dynamics simulation (inner iteration).

Nonlinear programming methods may be largely classified in two groups: local and global methods. Local methods are designed to generate a sequence of solutions, using some type of pattern search or gradient and Hessian information that will converge to a local optimum. However the NLP arising from the application of the control vector parameterization method are frequently multimodal (i.e. presenting multiple local optima), due to the highly nonlinear nature of the dynamics [41]. In this scenario, the initial guess may be located in the basin of attraction of a local minimum. This may be easily assessed by solving the problem from different initial guesses (multistart). In fact, this may be regarded as the first global optimization strategy. However experience demonstrates that there is no guarantee of arriving to the global solution, even starting from a large number of different initial guesses, and becomes computationally too expensive as illustrated in the examples considered in [10,42] and later in this work.

Over the last decade a number of researchers have proposed different techniques for the solution of multimodal optimization problems. Depending on how the search is performed and which information they are exploiting the alternatives may be classified in two major groups: deterministic and stochastic.

Global deterministic methods [43] in general take advantage of the problem’s structure and guarantee global convergence for some particular problems that verify specific smoothness and differentiability conditions. A number of works have recently approached the solution of dynamic optimization problems using convex relaxations or branch-and-bound strategies [42,44,45]. Although very promising, the necessary conditions for these methods to be applicable may not be guaranteed for the cases of interest and the computational cost may become prohibitive, particularly as the number of decision variables and the simulation cost increase.

The main drawbacks of global deterministic methods have motivated the use of stochastic methods that do not require any assumptions about the problem’s structure. They make use of pseudo-random sequences to determine search directions toward the global optimum. This leads to an increasing probability of finding the global optimum during the run time of the algorithm, although convergence may not be guaranteed. The main advantage of these methods is that, in practice, they rapidly arrive to the proximity of the solution.

The most successful approaches lie in one (or more) of the following groups: pure random search and adaptive sequential methods, clustering methods or metaheuristics. Metaheuristics are a special class of stochastic methods which have proved to be very efficient in recent years. They include both population (e.g., genetic algorithms) or trajectory-based (e.g., simulated annealing) methods. They can be defined as guided heuristics and many of them try to imitate the behavior of natural or social processes that seek for any kind of optimality [46]. Some of these strategies have been successfully applied to the dynamic optimization of bioprocesses [10].

Despite the fact that many stochastic methods can locate the vicinity of global solutions very rapidly, the computational cost associated to the refinement of the solution is usually very large. In order to surmount this difficulty, hybrid methods and metaheuristics that have been recently developed which combine global stochastic methods with local gradient based methods in two phases [47] or in several phases as in the scatter search based method eSS [23,48].

Finally, knowing that global optimization methods become prohibitively expensive with an increasing number of decision variables, a control refining technique has been used so as to obtain smoother control profiles. This technique consists of performing successive re-optimizations with increasing control discretization level. A detailed description of the mesh refining approach used is presented in [49]. The main steps are the following: 

· Step 1: The problem is solved using a coarse control discretization level (for example, 5−10) with the hybrid optimization method.

· Step 2: The best solution found is transformed by multiplying the discretization level by for example 2−4 and the result is employed as the starting point for the local method.

· Step 3: Step 2 is repeated until the established number of refinements has been achieved.

## Results and Discussion

It is well known that spatio-temporal patterns appear in biology from the molecular level to the supra-cellular level [50]. Some examples include, traveling pulses of action potentials in neural fibers [51], waves in cardiac tissues in the heart [27,28], aggregation of multicellular organisms, animal aggregates, etc [19]. Experiments show that simple chemical reactions and some elementary interactions can lead to the formation of complex spatio-temporal patterns that are sensitive to changes in the experimental conditions and may undergo complete rearrangement in response to particular stimuli [52].

The examples considered here are related to the computation of such stimuli which will originate a given desired pattern. The first example is related to the bacterial chemotaxis process while the second, the FitzHugh-Nagumo model, provides a qualitative description of some physiological processes, such as the neuron firing in the brain or the heart beat.

### Case Study I: Bacterial chemotaxis

Some types of cells are highly motile, they are able to sense the presence of chemical signals (chemoattractants) and guide their movement in the direction of the concentration gradient of these signals [53]. This process, called chemotaxis, has a role in diverse functions such as the sourcing of nutrients by prokaryotes, the formation of multicellular structures, tumor growth, etc. Therefore being of the highest interest not only to elucidate the mechanisms of the process to develop predictive models, but to use those models to externally control the process in a particular desired way.

The chemotaxis of the bacteria Escherichia coli is one of the best understood chemotactic processes. These bacteria, under given stress conditions, secrete chemoattractants. Other cells respond to these secreted signaling molecules by moving up their local concentration gradients and forming different types of multicellular structures [54].

The modeling of bacterial chemotaxis has received major attention during last decades. In contrast, only some works by Lebiedz and co-workers [19,20] consider the external manipulation of the process. These authors made use of a combination of the multiple shooting approach with a local optimization method to solve the problem reporting some difficulties due to the presence of local optima and the large computational costs associated. This work addresses the same problem, offering a detailed analysis of the presence of local solutions and proposing the use of global optimization methods to deal with its multimodal nature.

#### Mathematical model

The model under consideration describes the bacterial chemotaxis in a closed long thin tube containing a liquid medium with a cell culture of E. coli and the chemoattractant species which is produced by the cells themselves. The two components (bacteria and chemoattractant) may be described by a coupled reaction-diffusion system of PDEs which, in its 1D version, reads as follows [20]:

(17) $$\frac{\partial z}{\partial t}=D\frac{\partial^{2}z}{\partial \xi^{2}}-\mu \frac{\partial }{\partial \xi }\left(\frac{z}{{\left(1+c\right)}^{2}}\frac{\partial c}{\partial \xi }\right)$$

(18) $$\frac{\partial c}{\partial t}=\frac{\partial^{2}c}{\partial \xi^{2}}+\frac{z^{2}}{1+z^{2}}$$

with boundary and initial conditions of the form:

(19) $${\left(\frac{\partial z}{\partial \xi }\right|}_{\xi =0}={\left(\frac{\partial z}{\partial \xi }\right|}_{\xi =L}={\left(\frac{\partial c}{\partial \xi }\right|}_{\xi =0}=0$$

(20) $${\left(\frac{\partial c}{\partial \xi }\right|}_{\xi =L}=0$$

(21) z(ξ,0)=1;c(ξ,0)=0

where *z*(*ξt*) and *c*(*ξt*) represent the cell density and the concentration of the chemoattractant, respectively. *D* denotes the diffusion coefficient with a value of 0.33 while the model parameter *μ *is set to 80 - parameter values were taken from [20]-. The system is defined over the spatial domain $V=\left\{0\leq \xi \leq L\right\}$, with *L *= 1 being the tube length. The coupling between the nonlinear and diffusion terms in this process leads to different spatial patterns (aggregation of cells at given spatial regions) as a response to given perturbations (for instance changes in the initial or in boundary conditions) as shown in [20]. Some examples of cell aggregation patterns in a real chemotatic process can be found in [54].

#### Formulation of the optimal control problem

The objective is to externally manipulate the system so as to achieve a particular cell distribution. With this aim, a non-zero chemoattractant flux is introduced in the boundary *ξ *= *L*, resulting into:

(22) $$\frac{\partial c(L,t)}{\partial \xi }=u(t)-c(L,t)$$

Experimentally this can be achieved introducing in that boundary a semi-permeable membrane (impermeable to the cells but permeable to the chemoattractant). The boundary chemoattractant flux is controlled by fixing the concentration of this chemical species, *u*, in an external reservoir. Equation (21) indicates that the chemotractant flux entering/leaving the system is proportional to the difference between the concentrations at boundary *L* and at the external reservoir. We assume in this work that the control variables *u* may be modified instantaneously between two values in the range *u*∈[0,1].

As in [19] we will consider, in this work, two desired cell distributions: a Gaussian profile $z_{T,1}(\xi )=2.2\left(\exp\left(-25{\left(\xi -0.5\right)}^{2}\right)+0.1\right)$ and a constant profile *z*_*T*,2_(*ξ*) = 1. The optimal control problem may be then formulated as follows: *Findu*( *t*) *within the interval t *∈[0,1] *so as to minimize the deviation of the cell density as compared to the desired spatial distribution*. This is mathematically formulated as to find:

(23) $$\underset{u}{\min}J_{k}:=\frac{1}{2}\overset{L}{\underset{0}{\int }}{\left(z(\xi ,t_{f})-z_{T,k}(\xi )\right)}^{2}d\xi$$

where *k *= 1,2 represents the desired Gaussian and constant profiles, respectively. In order to numerically compute the integral term in (22), the spatial domain is discretized into *n*_*ξ*_equidistant points so that instead of (22) the following expression will be employed:

(24) $$\underset{u}{\min}J_{k}:=\frac{L}{2n_{\xi }}\overset{n_{\xi }}{\underset{j=1}{\sum }}{\left(z_{j}(t_{f})-z_{T,\text{kj}}\right)}^{2}.$$

Note that the summation extends over all the discretized points. The optimal control problem (23) is subject to: 

· The system dynamics described by Equations (16)-(18), (20) and (21).

· Bounds on the control variable 0≤u(t)≤1.

The sub-cases will be referred to as OCP1 (for the Gaussian distribution) and OCP2 (for the constant profile).

#### Results

##### *Simulation*

The finite difference method is employed in this case study to numerically compute the solution of system (16)-(21). Usually, in highly nonlinear systems as the one considered here, the spatial discretization level as well as the order of the finite difference formula play a central role in the computation of an accurate numerical solution. In order to avoid numerical solutions with no physical meaning (spurious solutions), a comparison among different schemes was performed.

**Figure 1:**
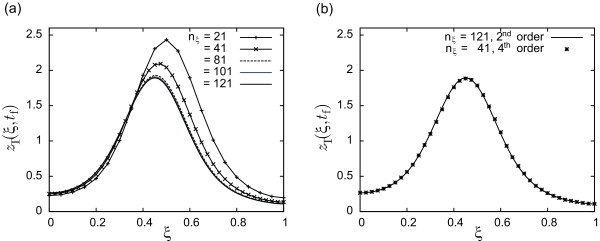
Analysis of simulation results, in terms of final time cell distribution as a function of (a) the spatial discretization level and (b) the order of finite differences formula.

Figure 1(a) presents the final time cell density distribution for a given control profile using different number of discretization points *n*_*ξ*_. From the figure, it is clear that using a low number of discretization points may result into large simulation errors thus leading to wrong conclusions about optimality. Note also that the solution seems to converge for *n*_*ξ *_> 101. On the other hand, one may also consider increasing the order of the finite differences formula and check whether it has a direct impact on the number of discretization points required to accurately represent the system dynamics. Figure 1(b) shows the comparison between using a second order formula with *n*_*ξ *_= 121 and fourth order formula with *n*_*ξ *_= 41. Since the results are almost indistinguishable, fourth order formula with *n*_*ξ *_= 41 is selected for optimization purposes as it provides the best compromise between accuracy and efficiency.

##### *Solution with a multistart approach*

A multistart strategy of a sequential quadratic programming method (FSQP, [55]) is used to simultaneously analyze the problem multimodal properties (for the selected control vector parameterization conditions) and the type of interpolation that seems to be more adequate for each case.

As explained in the “Numerical methods” section, in the control vector parameterization method the process duration is divided into a number of elements (discretization level). As a first approximation we selected a discretization level *ρ *= 7 and piecewise constant (PC), i.e. zero order polynomials, and piecewise linear (PL), i.e. first order approximations for the control variable. Both cases were solved using, as initial guesses, 300 randomly generated initial control profiles. To do so matrices of dimension 300× *ρ*, were generated within the lower and upper bounds using the MatlabⒸ function rand. The FSQP method was launched from each of the initial guesses until convergence tolerance 10^−5^ is achieved.

**Figure 2:**
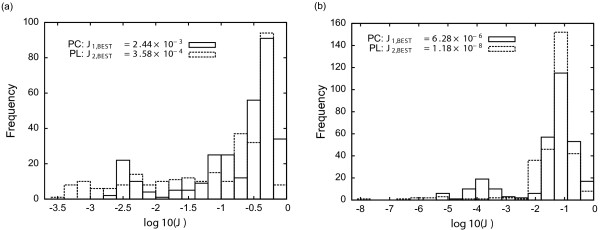
**Histograms of solutions for the multistart of FSQP for the chemotaxis related examples.** Results obtained from 300 runs from randomly generated initial control profiles. A comparison of optimal solutions obtained by means of *ρ *= 7 piecewise constant and linear control interpolations is presented. For both OCP1 **(a)** and OCP2 **(b)** the best reported value was obtained with piecewise linear interpolation.

The corresponding histograms of solutions are presented in Figure 2(a) for OCP1 and 2(b) for OCP2. The computational costs vary from one multistart to the other in a range of a few seconds to 6 min (in an Intel®; Xeon®; 2.50 GHz workstation using Matlab R2009b under Linux 32-bit). The total time employed in the 300 optimizations was around 250 min.

Let us analyze the results. First depending on the initial guess for the control, different solutions, with different objective function values, are obtained. Therefore the problem is multimodal and several orders of magnitude in *J* separate the best and the worst solutions. The use of PL polynomials for the control led to an order of magnitude improvement in OCP1 in comparison to the use of PC polynomials. The improvement in OCP2 is even larger. Therefore, in the following, the focus will be on PL polynomials. In addition note that most of the times the local solver converged to solutions with *J* values which are orders of magnitude larger than the best solution found. In both OCP1 and OCP2 cases the best solution was obtained only once in the 300 runs. From this analysis we can conclude that local solvers are not suitable for this problem and global methods must be employed.

##### *Solution with a hybrid technique*

To avoid getting trapped in suboptimal solutions, the use of global optimization methods is suggested. As mentioned previously the NLP solver eSS has proved to efficiently deal with a wide range of optimization problems. Therefore it has been chosen as the global NLP solver for this problem.

As in the multistart approach, a discretization level *ρ *= 7 with piecewise linear controls was employed. In order to check for the robustness of the NLP solver 10 optimizations for each of the optimal control problems have been performed. The results are summarized in Table 1. Note that the dispersion of these results is orders of magnitude lower than in the multistart cases and the mean value of the hybrid approach is comparable to the best value obtained with the multistart. For the case of OCP1 the value $J_{1,\text{BEST}}=2.59\times 10^{-4}$ is achieved in around 400 s while for OCP2 the optimal control profile found lead to $J_{2,\text{BEST}}=2.92\times 10^{-9}$ in 500 s. Note that none of the multistarts were able to reach those values. In fact a reduction of a 28% was obtained for *J*_1,*BEST*_, while *J*_2,*BEST*_ was improved by one order of magnitude. Also the time required to reach those solutions is much lower as compared with the total time of the multistarts.

**Table 1:** Optimization results for the chemotaxis case after 10 runs with eSS


	**Best value**	**Mean value**	**Worst value**
OCP1	2.59×10^−4^ (-3.59)	5.60×10^−4^ (-3.25)	2.39×10^−3^ (-2.62)
OCP2	2.92×10^−9^ (-8.53)	4.11×10^−8^ (-7.38)	1.49×10^−7^ (-6.83)

The values between parenthesis correspond with ***log***_10_(*J*).

##### *Solution with control refinement*

The best optimal control profiles obtained in the previous step (*ρ *= 7) are now refined (*ρ *= 14). The FSQP solver is employed to compute the solution of the optimization problem.

**Figure 3:**
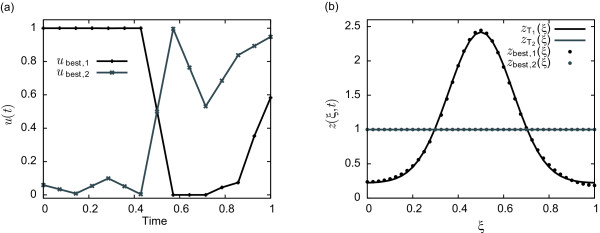
**(a) Optimal control profile obtained by the hybrid (*ρ*=14), linear interpolation) technique.(b)** Cell density distribution at final time.

For the OCP1, the hybrid approach with control refining allowed us to arrive to $J_{1,\text{BEST}}=2.36\times 10^{-4}$ with 15 s of extra computational effort. Note that an improvement of around an 8 % on the objective function value was achieved. On the other hand, when considering OCP2 the objective function value was improved by one order of magnitude $J_{1,\text{BEST}}=1.63\times 10^{-10}$ when refining the control up to *ρ *= 14. The mean relative error between the optimal control solution and the desired profile is lower than 4% for OCP1 and 1.4×10^−3^*%* for OCP2. Therefore both objectives are achieved with satisfactory accuracy and no further refinement will be performed. To illustrate this fact, the optimal control profiles and the corresponding cell density distributions are depicted in Figure 3(a) and 3(b), respectively.

### Case Study II: The FitzHugh-Nagumo problem

**Figure 4:**
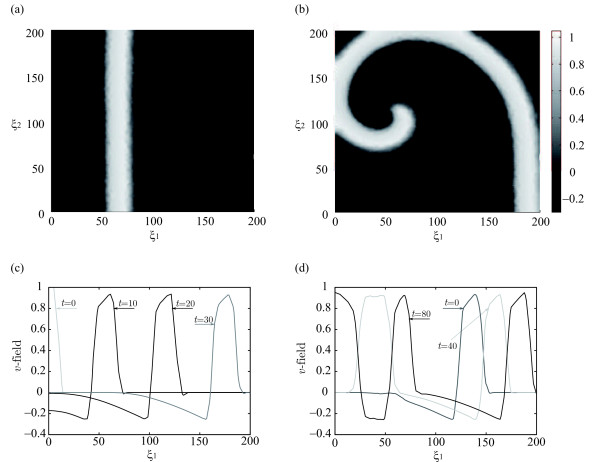
**The figures at the top show two snapshots of the*v*-field for the FHN system corresponding to (a) the front behavior and (b) the spiral behavior.** The figures at the bottom represent the *v*-field cross section at *ξ*_2_ = 100 and at different times corresponding to **(c)** the front behavior and **(d)** the spiral behavior.

Some physiological processes, such as the heart beating or the neuron firing, are related to electrical potential patterns. Their normal operation is associated to the formation of a traveling plane wave which spreads all over the tissue. Figure 4(a) shows a snapshot of this behavior while Figure 4(c) represent the cross section of the front at different times. Under certain circumstances, such as the presence of an obstacle in the cardiac tissue, the plane front can break leading to spiral wave formation as illustrated in Figure 4(b) (snapshot of the spiral behavior) and 4(c) (cross section at different times) [56]. This class of behavior is related to neurological disorders or cardiac dysfunctions such as arrhythmia and can lead, in case the spiral breaks, to more serious problems like fibrillation.

Due to the obvious necessity of preventing and/or controlling such undesirable behaviors, many research efforts have been devoted to the modeling of such processes. Particularly successful was the one developed by Hodgkin and Huxley [51] in early 50’s, able to predict the periodic, quasiperiodic and chaotic responses of the action potential in sinusoidal current stimulated giant squid axons. The complexity of that model led to the development of simplified versions, such as the one by FitzHugh and Nagumo [27,28].

It is worth mentioning that the control and stabilization of spatio-temporal fronts in biological system, and in particular the FHN system, has been successfully approached in the literature -see [25,57-59] and references therein-. Most of these works made use of electric fields of moderate intensity, computed through given feed-back control logics to attain the desired objective. However, to our knowledge, there is no previous works on the dynamic optimization of the FHN system. This work proposes the solution of a related dynamic optimization problem to calculate the stimulus that drives the system back to the desired behavior, in this case a traveling plane wave. Remark that the optimal dynamics may be then embedded into a feed-back control loop, for instance introducing the optimal solution into a model predictive control approach.

#### Mathematical model

In this work, we consider a 2D version of the FHN model. The system is defined over the square spatial domain $V=\left\{0\leq (\xi_{1},\xi_{2})\leq 200\right\}$ with the boundary B being the sides of the square, this is $B=(\xi_{1},\xi_{2})/(\xi_{1}=0\text{and}\xi_{1}=200,\forall \xi_{2}\in [0,200]),(\xi_{2}=0\;\text{and}\;\xi_{2}=200,\forall \xi_{1}\in [0,200])$. The model equations are [56]:

(25) $$\frac{\partial v}{\partial t}\;=\;\left(\frac{\partial^{2}v}{\partial \xi_{1}^{2}}\;+\;\frac{\partial^{2}v}{\partial \xi_{2}^{2}}\right)\;+\;f(v,w)\;+\;u;f(v,w)=(\alpha -v)(v-1)v-w;$$

(26) $$\frac{dw}{dt}=g(v,w);\;g(v,w)=\varepsilon (\gamma w-\delta -\beta v);$$

with boundary conditions:

(27) $${\left(\frac{\partial v}{\partial n}=0\right|}_{B}$$

In Equations (24)-(26), *v* (fast variable) is related to the membrane potential and is known as the activator while *w* (slow variable), the inhibitor, collects the contributions of ions such as sodium or potassium to the membrane current [50]. *ε *denotes the ratio between time scales for the activator and inhibitor kinetics. The parameters *α*∈(0,1), *βγ* and *δ* are non negative. The control inputs, related to low intensity currents, are collected in the term *u*. Finally, in Eqn. (26), **n **indicates a unit vector pointing outwards the surface. In this case study, the initial conditions take the form:

(28) $$v_{0}=\left\{\begin{array}{c} 1\;\text{if}\;0\leq \xi_{1}\leq 10\\ 0\;\text{if}\;10\leq \xi_{1}\leq 200 \end{array}\right)$$

(29) $$w_{0}=0,\;\forall \xi_{1},\xi_{2}$$

By setting the parameters *α *= 0.1, *ε *= 0.01, *β *= 0.5, *γ *= 1 and *δ *= 0, the solution of system (24)-(28) is a traveling plane front as the one shown in Figure 4(a). The FHN model is also able to capture the phenomenon related to cardiac arrhythmia illustrated in Figure 4(b). Such solution is obtained by resetting the superior half plane at a given time instant (i.e., the plane front is broken from *ξ*_2_ = 100 to *ξ*_2_ = 200).

The finite element method with a grid of around 2300 points has been employed to solve the boundary value problem (24)-(28). Coarser grids result into a front-type solution with low resolution while finer grids do not alter the solution. Note that, since two state variables are considered, such grid implies solving around 4600 ODEs which, for optimization purposes, is computationally involved. In order to overcome such limitation an accurate reduced order model derived by using the POD technique will be developed.

#### Reduced order model

As mentioned previously, the POD technique will be employed to obtain the reduced order model. In this methodology, five steps can be distinguished: 

· Obtain a set of snapshots representative of the system behavior

· Obtain the POD basis

· Decide how many basis will be employed in the projection

· Project the model equations (24)-(28) over the selected POD basis

· Solve the resulting ODE set

##### *Snapshots computation:*

This is a critical point in the POD technique. In order to obtain an accurate reduced order model, the snapshots must be representative of the system behavior. Unfortunately, there is no systematic approach to decide the conditions that better represent the system behavior. However, the idea is to capture as much information as possible from a limited set of snapshots that may be obtained either through simulation of the original model or through appropriate experimental setups.

In our case all the snapshots were obtained from simulation of system (24)- (26). The first set of snapshots aimed to capture the front-type behavior, to that purpose the simulation started with initial conditions (27)- (28) setting the control *u *= 0 and lasted for *t *= 200 taking one snapshot each *Δt *= 10. A second set was computed to capture the spiral behavior, first such behavior was induced by resetting the superior half plane at a given instant then snapshots have been taken each *Δt *= 10 till *t *= 200 with *u *= 0. Finally, 15 extra simulation experiments were performed to capture the effect of the control variable. In each of these experiments initial conditions correspond with the spiral behavior (see Figure 4(b)) and time was divided into 10 equally spaced segments with a duration of *Δt *= 6. During each time segment a randomly generated control input *u *∈ [−1,1] was applied.

##### *POD basis computation:*

Once the snapshots are available they are employed to construct the kernel *R*( *ξξ*^*′*^) as in Eqn (10). In fact two kernels (*R*_*v*_(*ξξ*^*′*^) and *R*_*w*_(*ξξ*^*′*^)) will be constructed from the snapshots of the state variables *v* and *w*, respectively. Then the POD basis are computed by solving the integral eigenvalue problem (9). To that purpose, the mass matrix obtained from the application of the finite element method is exploited to numerically compute spatial integrals (for a detailed discussion see [60]). As a result of this step, two basis sets (*Φ*_*v *_=[*ϕ*_*v*1_*ϕ*_*v*2_,…,*ϕ*_*vn*_] and *Φ*_*w *_=[*ϕ*_*w*1_*ϕ*_*w*2_,…,*ϕ*_*wm*_]) are obtained.

##### *Number of POD basis employed to project:*

**Figure 5:**
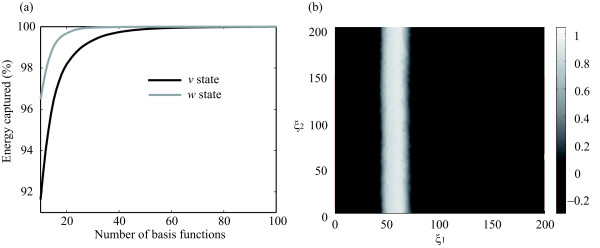
**(a) Energy captured by the POD basis.(b)** Reduced order model solution for the FHN system (front behavior).

This will determine the dimension of the reduced order model. The criteria used to compute the number of POD basis is based on the energy captured by them -see Eqn (11)- which is represented in Figure 5(a). A 99.95*%* of the energy is enough to accurately represent the system, therefore, 85 and 28 PODs basis will be employed, respectively, in the projection of state variables *v* and *w*.

##### *Projection of the PDE system:*

As explained in section *numerical methods for simulation* projection is carried out by multiplying the original PDE system by the POD basis and integrating the result over the spatial domain V. Note that the finite element structure may be also exploited in this step [60]. In this case this procedure leads to the following ODE system:

(30) $$\frac{dm_{v}}{dt}=P_{A}m_{v}+F_{A}+U_{A};$$

(31) $$\frac{dm_{w}}{dt}=G_{A};$$

where

(32) $$\begin{array}{l} P_{A}=\int_{V}\Phi_{v}^{T}\left(\frac{\partial^{2}\Phi_{v}}{\partial \xi_{1}^{2}}+\frac{\partial^{2}\Phi_{v}}{\partial \xi_{2}^{2}}\right)d\xi ;\\ F_{A}=\int_{V}\Phi_{v}^{T}f(v,w)d\xi ;\\ U_{A}=\int_{V}\Phi_{v}^{T}\text{ud}\xi ;\\ G_{A}=\int_{V}\Phi_{w}^{T}g(v,w)d\xi \end{array}$$

Initial conditions are also projected as follows:

(33) $$m_{v0}=\int_{V}\Phi_{v}v_{0}d\xi ;\;m_{w0}=\int_{V}\Phi_{w}w_{0}d\xi$$

As a result a system with 113 ODEs (more than 40 times lower than the classical finite element method) is obtained.

##### *Solution of the ODE set:*

Finally, the solution of (29)- (31) is computed by a standard initial value problem solver. Figure 5(b) represents spatial distribution of the *v* state variable at a given time instant computed using the reduced order model. Note that this solution approximates with satisfactory accuracy that one obtained using the finite element method with a grid of around 2300 points - see Figure 4(a) -.

#### Optimal control problem formulation

**Figure 6:**
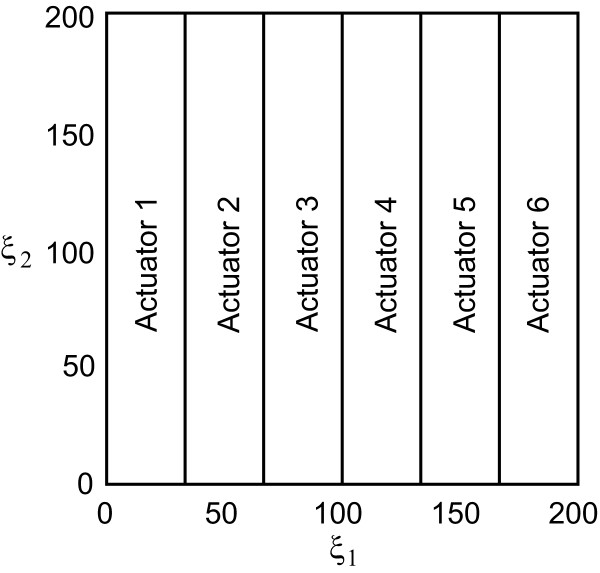
Distribution of the six actuators over the spatial domain.

The aim of this section is to design an open-loop optimal control policy (*u*) able to drive the spiral behavior back to the plane front. For practical reasons, it is assumed that only a limited amount of actuators ( *n*_*a *_= 6) are available. In this regard, as shown in Figure 6 the spatial domain is divided into six vertical bands which correspond to actuators supplying spatially independent currents.

The optimal control problem is then formulated as follows: *findu*_*k*_(*t*) *withk *= 1,…,6 *within t *∈[0,60] *so as to drive the system from the spiral behavior to the desired front pattern v*_*T*_(*ξ*_1_,*ξ*_2_)* represented in Figure*4*(a).* Mathematically this can be expressed as to find:

(34) $$\underset{u}{\min}J;\;\text{with}\;J=\frac{1}{n_{\xi }}\overset{n_{\xi }}{\underset{i=1}{\sum }}{\left(v_{i}(t_{f})-v_{\text{Ti}}\right)}^{2}$$

Subject to: 

· The reduced order model dynamics (29)-(31)

· Bounds on the control variables, $-1\leq u_{k}(t)\leq 1$.

#### Results

Similarly to the previous case, a multistart approach of the FSQP method was selected to study the possible multimodal nature of the problem. As a first approximation we selected a discretization level *ρ *= 10 and piecewise constant control. 250 randomly generated initial control profiles were used to launch FSQP method. To do so matrices of dimension 250×6 *ρ*, were generated within the lower and upper bounds using the MatlabⒸ function rand.

**Figure 7:**
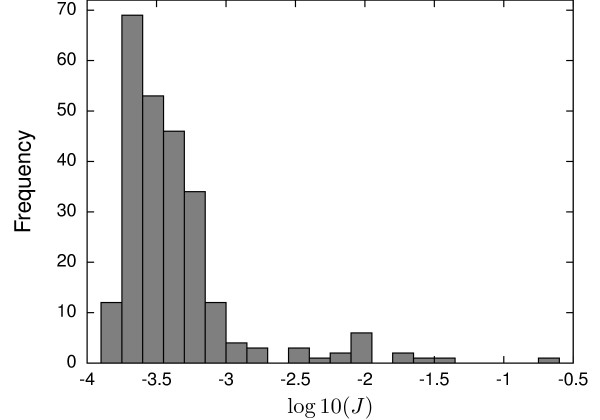
**Histogram of solutions for the multistart of the FHN system (**$J_{\text{BEST}}=1.44\times 10^{-4}$**).**

Results obtained are summarized in Figure 7. A quick view to this figure shows us two things: first, the presence of several suboptimal solutions and second, the huge distance, more than three orders of magnitude in the objective function values, between the worst and the best solutions. Note also that less than 5 % of the times the local solver converged to values close to the global solution.

**Figure 8:**
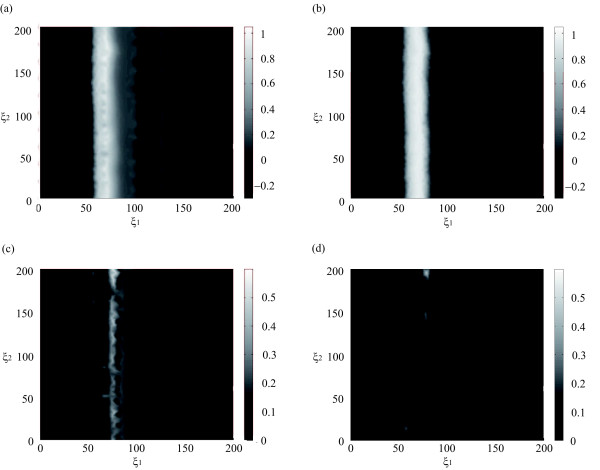
**Figures (a) and (b) represent the*v*-field final time spatial distribution after the implementation of an intermediate control profile from the multistart and the global optimal control profile, respectively.** Figures **(c)** and **(d)** represent the absolute error between the desired profile (Figure 4 **(a)**) and the profiles obtained with the optimal control.

In order to illustrate the effects of falling into suboptimal solutions, one of the locally optimal control profiles (with *log*_10_(*J*) = −2.5) was applied to the system. Figure 8(a) and (c) represent the resulting *v*-field spatial distribution at final time and the absolute error with respect the desired profile, respectively. The front obtained is not only larger than the desired one but also three new (undesirable) fronts appear from *ξ*_1_>100. The use of the hybrid technique is thus suggested so as to achieve the best possible solution in reasonable computational costs.

As in the chemotaxis case study we choose here the NLP solver eSS to compute the optimal solution. In order to compare the results with those of the multistart, the control discretization was fixed to *ρ*_1_ = 10, i.e. 60 decision variables and 10 optimization were performed to check the robustness of the solver. The best optimal profile found lead to a cost function value of $J_{\text{BEST}}=1.44\times 10^{-4}({\text{log}}_{10}(J_{\text{BEST}})=-3.76)$ which coincides with that of the multistart best solution while the mean and the worst cases over the 10 runs were, respectively, $J_{\text{mean}}=2.53\times 10^{-4}({\text{log}}_{10}(J_{\text{mean}})=-3.60)$ and $J_{\text{worst}}=4.56\times 10^{-4}({\text{log}}_{10}(J_{\text{worst}})=-3.34)$. It is important to highlight that the computational time required to arrive to such a value was several orders of magnitude lower as compared with the total time of the multistart approach.

From that solution the FSQP method was used with a refining on the control discretization level (*ρ*_2_ = 20), resulting into a NLP problem with 120 decision variables. After the optimization, a value of the objective function of $J_{\text{BEST}}=1.32\times 10^{-4}$ was achieved, i.e., an improvement of around a 6%. This optimal solution obtained using the reduced order model (29)- (31) was implemented in the “real” (finite element) process. The resulting *v*-field spatial distribution at final time and the absolute error with respect the desired profile (Figure 4(a)) are represented in Figures 8(b) and (d), respectively. The larger differences now concentrate in those regions where the front is steeper while, in the rest of the spatial domain, errors are negligible.

**Figure 9:**
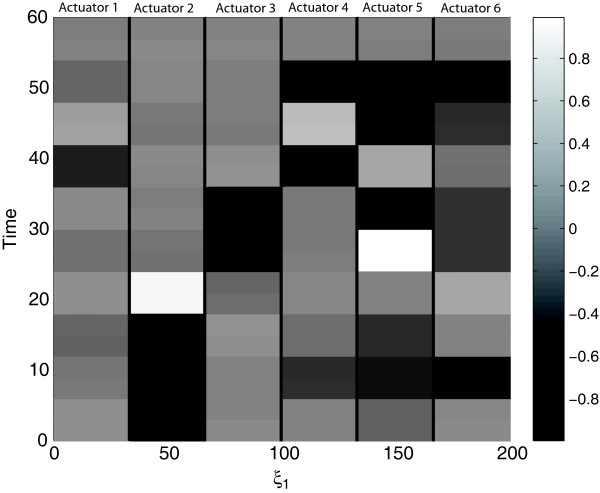
Heat map of the optimal control profiles for the FitzHugh-Nagumo problem.

Finally, the optimal control profiles for the spatially independent currents are represented in Figure 9.

## Conclusions

The combination of advanced numerical optimization techniques with reduced order based models enables the possibility of efficiently solve dynamic optimization problems related to complex distributed biological systems.

The simulation of non-linear and distributed models by means of typical spatial discretization techniques is usually computationally intensive. In addition, non-linear dynamics often induce multimodality in the associated optimization problems. Therefore calling for global optimization methods which often require a large number of model simulations. These pose important constraints to the solution of dynamic optimization problems related to distributed biological systems.

This work has shown, with two illustrative examples, how these difficulties can be surmounted with the following procedure: 

· Use spatial discretization techniques, such as the finite differences or the finite element method, to handle process simulation under different control conditions and generate the snapshots, i.e., numerical values of the spatio-temporal evolution of the state variables.

· Use these snapshots to obtain a more efficient dynamic representation (reduced order model) via the proper orthogonal decomposition approach. Such reduced order model will be employed instead of the complete model, in the following steps, to enhance the efficiency of the solution of the optimization problem.

· Solve the dynamic optimization problem with a coarse discretization and stepwise approximation of the control variables by means of a local NLP solver with a multistart approach (i.e. using multiple initial guesses). If and when the presence of multimodal objective function is confirmed from multistart local optimizations (typically involving 25-50 initial guesses), a hybrid stochastic-local optimization method such as the scatter search based approach should be used.

· Obtain smoother control profiles, if required, by means of a mesh refining technique or a piecewise linear interpolation of the control variables.

## Endnote

^a^ For the sake of clarity and without loss of generality, the vector field **x**( *ξ*, *t*) in Eqn (2) will be considered as a scalar *x*( *ξ*, *t*)

## Competing interests

The authors declare that they have no competing interests.

## Author’s contributions

All authors contributed to the conception and design of the work. JRB and EBC selected the numerical methods for optimization and case studies. CV and AA selected the numerical methods for simulation. CV and EBC performed the numerical computations. All authors contributed to the writing of the manuscript. All authors read and approved the final manuscript.
